# Dermatofibrosarcoma protuberans (DFSP) arising from a keloid scar – A case report

**DOI:** 10.1016/j.jpra.2018.09.002

**Published:** 2018-10-02

**Authors:** Joseph Ward, Joy Odili

**Affiliations:** Department of Plastic Surgery, St George's Hospital, St George's Hospital NHS Trust, Blackshaw Road, Tooting SW17 0QT, United Kingdom

**Keywords:** Case report, Dermatofibrosarcoma protuberans, Keloid scar, Latissimus dorsi myocutaneous flap

## Abstract

Dermatofibrosarcoma protuberans (DFSP) is a rare cutaneous sarcoma with an indolent early course that may be misdiagnosed for benign skin pathology. In this case-report we highlight an instance of DFSP arising from a keloid scar *de novo* and present a reconstruction with a local pedicled LD flap. We subsequently appraise the related literature and discuss the diagnostic challenges.

## Introduction

Dermatofibrosarcoma protuberans (DFSP) is a rare, locally invasive, low to intermediate-grade cutanaeous sarcoma arising from the dermis with an incidence of 0.8–4.5 cases/million/year.^1^, ^2^ First described by Darier and Ferrand-Drake in 1924^3^ as a “progressive recurring dermatofibroma”, it was more fully pathologically defined by Hoffman in 1925.^4^ Most often presenting as a slow-growing asymptomatic flesh-coloured nodule or plaque, DFSPs in the latter stages (2–5% of cases) may undergo rapid change, infiltrate deeply and metastasise. In this case report, we present an example of DFSP arising from a long-standing keloid scar, demonstrate an elegant pedicled regional flap reconstruction and review the related literature.

## Case history

**Figure 1:**
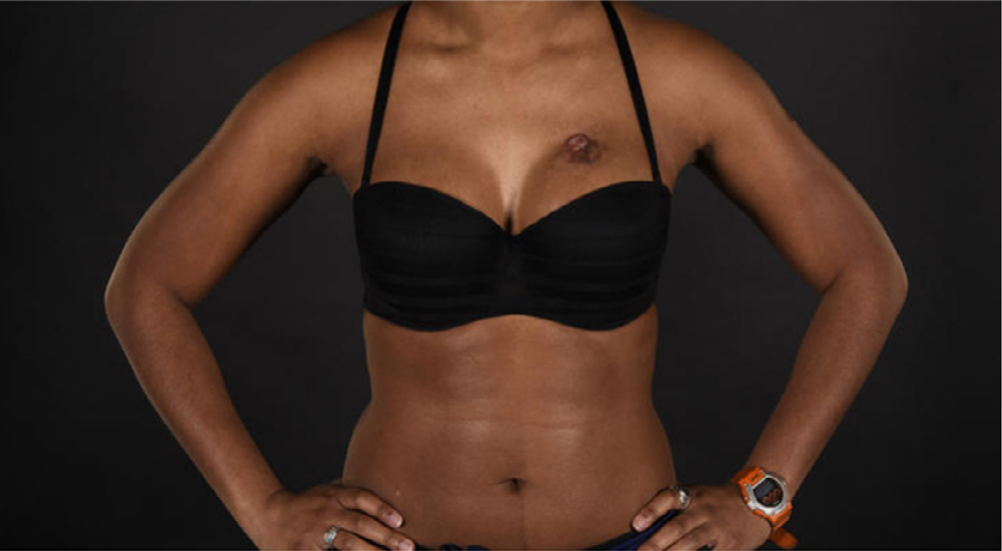
Pre-operative clinical photograph demonstrating nodular growth arising from pre-exisiting scar.

**Figure 2:**
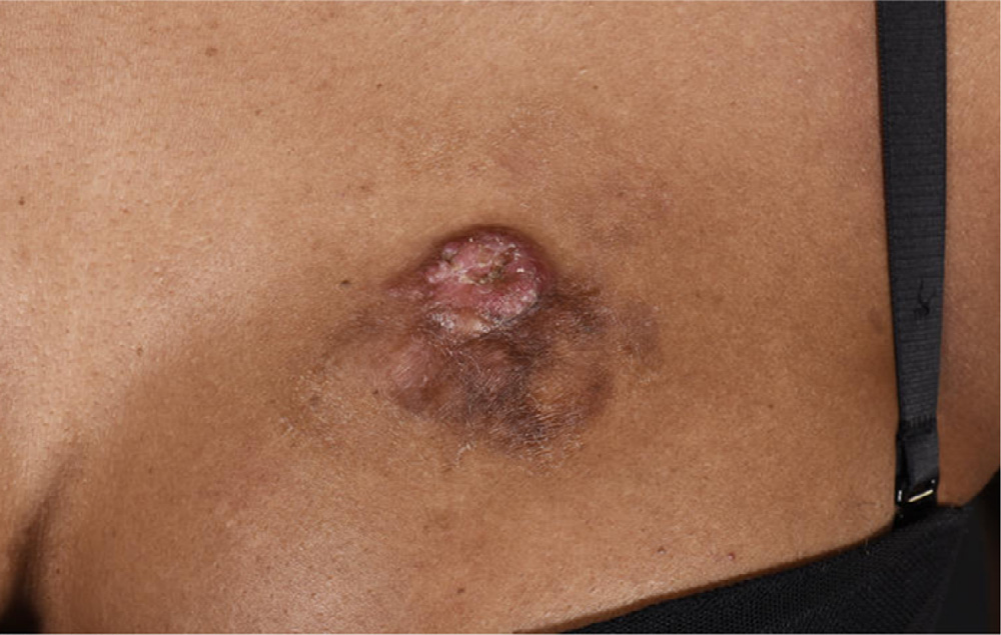
Higher power/close-up clinical photograph demonstrating lesion.

A 31-year-old female presented to the plastic surgery OPD with a painful, intermittently bleeding mass on her left anterior chest. Clinical history revealed a keloid scar present at the same site for over a decade resulting from a cat scratch during early adulthood. The patient clearly recalled that the wound on her chest had taken months to heal following the injury with the scar eventually becoming lumpy and keloid. The scar had previously been managed with steroid injections. The patient reported a recent change in the appearance of the keloid scar over the preceding year. She was otherwise fit and well. Clinical examination revealed a 3 × 2 cm fungating lesion at the superior pole of a partially involuted scar, with no associated palpable lymphadenopathy (see Figures 1 and 2).

**Figure 3:**
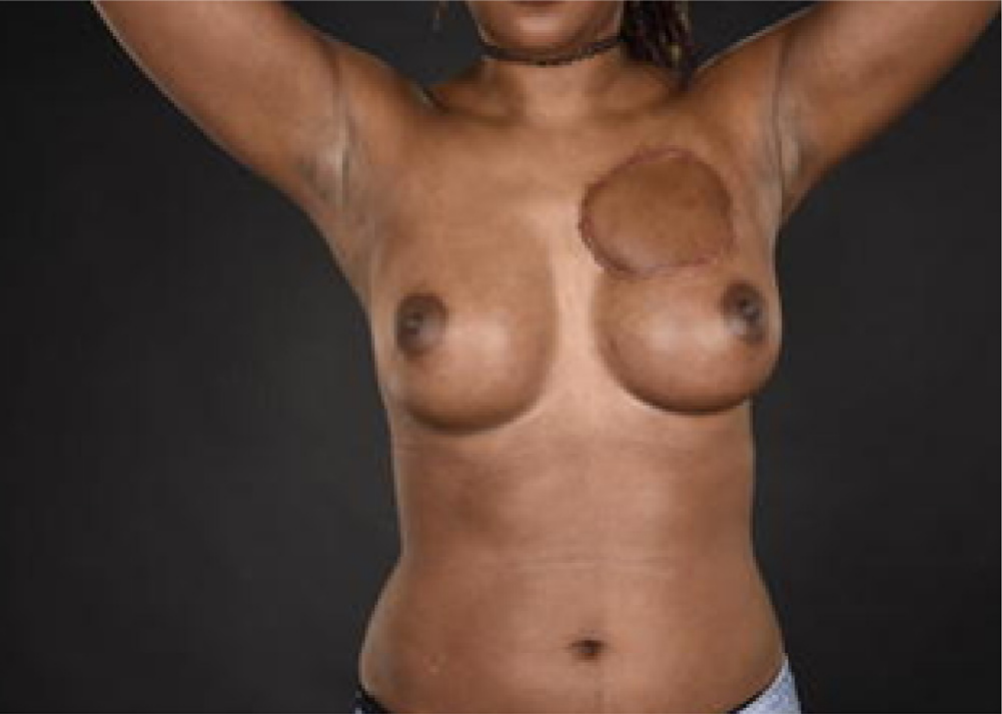
Final post-operative result with reconstruction using pedicled myocutaneous latissims dorsi flap. Flap raised through axilla to minimise donor site scarring.

An incisional biopsy demonstrated a spindle cell lesion infiltrating the dermis and subcutaneous fat with immunohistochemical features suggestive of dermatofibrosarcoma protuberans (DFSP). The patient was discussed at a specialist skin cancer MDT where recommendation for wide local excision was made. She underwent a staged surgical excision, with reconstruction carried out once histological margins were confirmed as clear and adequate. The defect was both wide and deep and the tumour was found to involve both breast tissue and pectoralis fascia. The defect was reconstructed using a pedicled latissimus dorsi myocutaneous flap (see Figure 3). The patient remains under follow-up with evidence of recurrence at 1-year post-op.

## Discussion

DFSPs represent 0.1% of all cutaneous malignancies and account for 2–6% of soft-tissue sarcomas.^5^, ^6^ In similarity to our case, the peak incidence is between 20 and 50 years of age^7^ and cohort studies demonstrate increasing age and African descent as incident risk factors.^2^ The distribution between sexes is equal and age-adjusted incidence is 1 per 100,000 individuals per year.^7^, ^8^ DFSPs show an anatomical predilection for the trunk (40–50%) but may otherwise commonly be identified on the proximal limbs (30–40%) and head and neck (10–15%).^6^, ^9^^,^^10^

DFSPs are most frequently diagnosed during their indolent phase when superficial, less than 5 cm in size, non-fixed and confined to the dermis. In view of their malignant potential with high-risk for local recurrence, surgery represents the mainstay of treatment for DFSP on diagnosis.^11^ Several studies have evaluated the role of conventional wide local excision vs. Mohs micrographic surgery (MMS) for management of DFSP demonstrating superiority for MMS. In the largest available comparative observational study, 79 patients were treated with either MMS or conventional surgery.^12^ During the 15-year study period, recurrence rates were 13.2% (95% CI: 4.4–28.1%, *n*=38) for conventional surgery against 0% (95% CI: 0–8.6%, *n*=41) for MMS. When the study data was pooled with all other available published studies this finding was confirmed with recurrence rates of 20.7%, 95% CI: 18.6–22.9%, *n*=1394) and 1.3% (95% CI: 0.5–2.8%, *n*=463), respectively. Such findings have been supported further by more recent systematic reviews^13^ and have led to treatment guidelines favouring MMS over conventional wide local excision particularly in sites where tissue preservation is paramount.^11^ Where MMS is unavailable a very wide peripheral margin of 3 cm should be taken alongside deep excision to a fascial plane. MMS is currently not available in our unit. Hence the patient underwent staged excision, proceeding to reconstruction once excision margins were clear histologically.

DFSPs have an excellent prognosis if completely and widely excised. Ten-year survival rates are high (>99%)^14^ with distant and regional metastases rare occurring in 1% and 6% of patients, respectively.^9^ Guidelines recommend prolonged follow-up of up to 10 years (6-monthly for 5 years and annually thereafter) to ensure any recurrence, which may often be delayed, is not missed.^11^ Systematic reviews have demonstrated that the mean time to recurrence is 68 months^13^ with the largest and most recent study demonstrating an annual recurrence rate of 0.6%, confirming delayed or late recurrence is not uncommon.^15^ DFSPs are radiosensitive tumours and radiotherapy can be offered for inoperable or recurrent disease.^11^, ^16^

The treatment of metastatic DFSP can be challenging given its nature as a chemo-resistant tumour. Anecdotally, methotrexate has been employed with limited evidence of efficacy.^17^ The tyrosine kinase inhibitor, Imatinib, has shown recent promise and targets PDG-signalling through inhibition of receptor phosphorylation of the PDGFβ receptor, ABL and KIT pathways.^18^ In light of the low incidence of DFSP and the only recent advent of Imatinib experience is limited but growing. The Imatinib Target Consortium Study B2225 evaluated 24 locally advanced or metastatic DFSP patients with the t (17;22)(q22;q13) (COL1A1;PDGFB) translocation – a marker of fibrosarcomatous transformation and demonstrated encouraging results with objective response rates of 45.9%^19^ A further clinical study, evaluating 31 DFSP patients with locally advanced or metastatic disease, treated in routine clinical practice, confirmed encouraging efficacy with 68% of patients undergoing a partial response, overall 5-year survival of 64% and a number of metastatic patients becoming amenable to potentially curative surgical resection after treatment.^20^ In the neo-adjuvant setting Imatinib also demonstrates promise.^11^

In our case, the DFSP arose from a pre-existing keloid scar. Reviewing the literature, there appears to be only one instance where DFSP transformation from a keloid scar has reported. This was in a 26-year-old black male with a history of multiple keloids who presented with a progressive right post-auricular neck lesion present since 10-years-of-age, which was then excised and histologically confirmed to be a keloid. The patient subsequently underwent re-excision of recurrence 8 years later where DFSP was demonstrated and unfortunately died 2 years later from widespread metastatic disease.^21^ The difficulty of discriminating DFSP from keloids is reflected in the literature with a number of authors highlighting that DFSP may masquerade or be misdiagnosed as a keloid.^22^ The risk is greatest where the lesion is small (<2 cm in diameter), unconnected with a clear history of trauma and exacerbated by the indolent nature of early DFSPs.^23^ Discriminating DFSPs from keloid scars can be a challenge clinically but should not be difficult pathologically. Characteristically, a DFSP will demonstrate diffuse dermal and fatty infiltration of CD34 +ve, Factor XIIIa +ve, stromelysin +ve tumour cells arranged in a storioform manner with a high mitotic rate. This contrasts with keloids that demonstrate significant collagen deposition, an absence of positivity for the above markers, an intact non-flattened epidermis and papillary dermis, as well as low vascularity and show α-SMA positivity. A particular hallmark for DFSP is the genetic t (17;22)(q22;q13) (COL1A1;PDGFB) translocation present in 90% of cases and can be sought in instances of diagnostic uncertainty.^11^, ^24^^,^^25^

We chose to reconstruct the defect for our patient with a pedicled latissimus dorsi flap - a flap commonly used for breast reconstruction. We chose this regional option for its reliability, ease of harvest and transposition as well as the ability to provide a large skin paddle leaving a cosmetic donor site hidden within the patient's bra strap. With this flap we were able to provide supple uniform tissue for her chest wall and replace the volume of breast tissue excised with a single flap. Alternative options considered for this area include a superiorly-based pedicled VRAM (although this would violate the abdominal wall), a pectoralis major turnover flap with skin grafting or free-flap reconstruction.

In summary, this case demonstrates how DFSPs are often mistaken for keloids, and highlights that DFSPs may arise from keloids *de novo*. It should be included in any differential diagnosis for apparently benign indolent fibrous skin lesions, particularly in younger patients. As a cutaneous sarcoma DFSPs should be managed aggressively in conjunction with a sarcoma or specialist skin multi-disciplinary team. Despite emerging oncological therapy, surgical excision represents the mainstay of treatment with good 5-year outcomes. Follow-up is advised given the predilection of delayed recurrence.

## Conflict of interest

The authors declare no conflict of interests.
